# Clinical Analysis of Ocular Parameters Contributing to Intraoperative Pain during Standard Phacoemulsification

**DOI:** 10.1155/2017/9463871

**Published:** 2017-04-12

**Authors:** Yong Koo Kang, Myung Jun Kim, Hong Kyun Kim, Bo Young Chun

**Affiliations:** ^1^Department of Ophthalmology, Kyungpook National University School of Medicine, Daegu, Republic of Korea; ^2^Department of Ophthalmology, Cheil Eye Hospital, Daegu, Republic of Korea

## Abstract

*Purpose*. To study the correlation between ocular parameters and subjective pain that patients perceived during phacoemulsification. *Methods*. Medical records of 142 patients who underwent standard phacoemulsification under topical anesthesia between March and August 2016 were retrospectively reviewed. The pain during phacoemulsification and 1 h after surgery was assessed and compared using a visual analog scale. In addition, demographic data, preoperative biometric parameters, and intraoperative surgical parameters were recorded. *Results*. Mean age of patients was 67.49 ± 12.50 years. The mean pain score was 2.26 ± 0.85 during phacoemulsification and 0.40 ± 0.69 postoperatively. Intraoperative pain was significantly associated with higher preoperative intraocular pressure (*β* = 0.220, *P* = 0.016), greater anterior chamber depth (*β* = 0.210, *P* = 0.028), and greater axial length (*β* = 0.181, *P* = 0.043). *Conclusions*. To reduce the subjective pain when patients have high preoperative intraocular pressure, large anterior chamber depth, or great axial length, supplementary procedures may be required.

## 1. Introduction

Phacoemulsification under topical anesthesia, first described by Fichman in 1992, has become the standard care for routine cataract surgery [1]. It allows rapid visual rehabilitation, causes less patient anxiety, and provides sufficient anesthesia while reducing the risk of serious complications such as globe perforation and retrobulbar hemorrhage associated with retrobulbar anesthesia [2, 3].

Patient cooperation is a must for the success and ease of phacoemulsification surgery under topical anesthesia. Perceived pain directly affects the patient's cooperation and has been reported to vary with age, sex, and whether the surgery is being performed for the first time [4, 5]. However, no information has been published on ocular parameters that contribute to intraoperative pain during phacoemulsification under topical anesthesia.

In this study, we analyzed parameters contributing to pain in patients having phacoemulsification and intraocular lens implantation under topical anesthesia alone.

## 2. Patients and Methods

Medical records were retrospectively reviewed after approval was received from the Institutional Review Board of Kyungpook National University Hospital. The review was conducted in accordance with the tenets of the Declaration of Helsinki.

Patients scheduled for elective phacoemulsification and intraocular lens implantation (IOL) with the Infiniti Vision System (Alcon, Fort Worth, TX, USA) were included. Because undergoing secondary eye surgery has been reported to cause more severe pain, only patients having their first operation were evaluated [6]. Cataract severity was graded by the Lens Opacities Classification System III [7]. All operations were performed in Kyungpook National University Hospital by the same surgeon (K.H.K.) between March and August 2016.

Patients who needed general anesthesia or were taking medications capable of affecting perceived pain were excluded from this study. Patients who had traumatic cataract and/or conditions likely to require vitrectomy and transscleral fixation of posterior chamber intraocular lens were also excluded.

Topical anesthesia (0.5% proparacaine HCl) eye drops were administered into the lower conjunctival sac three or four times in the 10 min preceding surgery. Clear corneal phacoemulsification was performed through a 2.2 mm incision using standard surgical parameters (Table 1). Cataract extraction comprised of continuous curvilinear capsulorhexis, phacoemulsification using divide-and-conquer or phacochop techniques, and aspiration of remaining cortical lens material followed by widening of the corneal tunnel to implant a hydrophobic acrylic intraocular lens. After the ophthalmic viscoelastic substance was removed, the wound was sealed by corneal stromal hydration.

## 3. Pain Assessment

**Figure 1:**
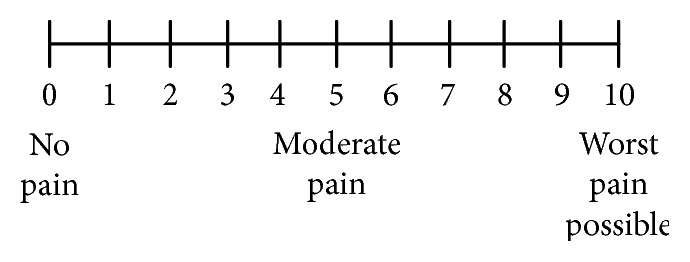
The visual analog scale of pain.

A third person assessed intraoperative and postoperative subjective pain using a pain visual analog scale (Figure 1) ranging from 0 (no pain) to 10 (worst pain imaginable) after surgery [8]. Patients were asked to score separately the pain during the procedure and the pain one hour after the procedure. All of our patients who underwent phacoemulsification received slit-lamp examination in our outpatient center one hour after the surgery to confirm the IOL position and cells of the anterior chamber cells. Thus, we assessed intraoperative pain immediately following the surgery in the surgery center and assessed postoperative pain in our outpatient center when doing slit-lamp examination.

## 4. Outcome Measures

The primary outcome measures at baseline were best-corrected visual acuity using the Snellen chart converted to the logarithm of the minimum angle of resolution (logMAR) and intraocular pressure (IOP) measured with the Goldmann applanation tonometry. Axial length (AL) and anterior chamber depth (ACD) were measured using partial coherence interferometry (IOLMaster, Carl Zeiss Meditec AG, Jena, Germany). A noncontact specular microscope (NSP-9900, Konan Medical Inc., Hyogo, Japan) was used to measure corneal endothelial cell density, and a rotating Scheimpflug camera (Pentacam, Oculus Inc., Wetzlar, Germany) was used to measure central corneal thickness.

The duration of surgery, recorded by the staff, was considered as the time from when the eye was covered with a drape to the extraction of the lid speculum. Ultrasound time, cumulative dissipated energy, and mean volume of balanced salt solution used were recorded. When adjuvant procedures were performed during surgery (e.g., insertion of capsular tension ring in zonular instability, intracameral injection of epinephrine hydrochloride 0.001% for mydriasis and carbachol 0.01% for miosis, and prophylaxis against increased IOP), these were also recorded.

## 5. Statistical Analysis

Statistical analyses were performed using SPSS statistical software 20.0 (SPSS Inc., Chicago, IL, USA). Paired *t*-test and the Pearson correlation coefficients were used to compare the pain score between intraoperative and postoperative periods. Univariate linear regression analysis was performed to compare intraoperative pain and clinical factors. A multiple linear regression analysis including variables with *P* values < 0.05 in the univariate analysis was performed to assess the effect of each variable on intraoperative pain score. For all statistical tests, a *P* value < 0.05 was considered significant.

## 6. Results

A total of 142 eyes of 142 patients were reviewed in this study. Their clinical characteristics are shown in Tables 2 and 3.

The mean pain score was 2.26 ± 0.85 during phacoemulsification and 0.40 ± 0.69 at 1 h after completion of the procedure, with a significant difference between the two periods (*P* < 0.001). The mean intraoperative and postoperative pain scores were significantly correlated (*β* = 0.710, *P* < 0.001).

Table 4 presents the results of univariate and multivariate linear regression analysis of the correlation between the clinical parameters and pain during phacoemulsification. Four parameters, preoperative IOP (*β* = 0.225, *P* = 0.007), AL (*β* = 0.255, *P* = 0.002), ACD (*β* = 0.202, *P* = 0.016), and cataract grade (*β* = −0.185, *P* = 0.028), were significantly associated with intraoperative pain in the univariate analysis. In the multivariate analysis, however, pain during phacoemulsification was significantly associated with higher preoperative IOP (*β* = 0.220, *P* = 0.016), larger ACD (*β* = 0.210, *P* = 0.028), and greater AL (*β* = 0.181, *P* = 0.043; *R*^2^ = 0.302).

## 7. Discussion

Phacoemulsification under topical anesthesia is preferred due to the safety and efficacy of this method as compared with other types of anesthesia. However, patient cooperation is directly related to perceived pain, and pain management may significantly influence the success of surgery and the occurrence of postoperative complications. Moreover, our results show that a patient who experienced more pain during surgery also has more postoperative pain, so the management of postoperative pain is also important in these cases.

O'Brien et al. reported that the highest mean pain score was at the phacoemulsification stage during cataract surgery procedures [9]. During the phacoemulsification, mobilization of the iris-lens diaphragm is postulated to cause discomfort during phacoemulsification cataract surgery. There are several variables related to iris-lens diaphragm mobilization. To reduce surgical pain, we should be aware of the pain-related preoperative and intraoperative parameters among the variables.

In our study, patients with higher baseline IOP had more pain during surgery. Higher baseline IOP could contribute to the elevation of IOP during cataract surgery and increase intraoperative pain. IOP fluctuations during phacoemulsification also could affect the elevation of intraoperative pain. Thus, it is essential to understand pharmacodynamics and widely used IOP control techniques, such as decreased bottle height or infusion pressure. Moreover, there are several reports of medications that lower IOP to prevent IOP spikes and to control IOP elevation [10, 11].

In addition, patients with larger ACD or greater AL had increased subjective pain during surgery. Hou et al. reported that in most patients pain was increased when the anterior chamber was extended by irrigation [12]. Particularly in eyeballs with long AL, whose sclera resistance is less than normal, distention of the anterior chamber is more likely. Although ACD is not always proportional to AL, there were positive correlations between ACD and AL in other researches and in our study (*β* = 0.432, *P* < 0.001) [13, 14]. Thus, these findings support our results that these patients tended to experience more pain intraoperatively.

Patients with lens-iris diaphragm retropulsion syndrome, first described by Wilbrandt and Wilbrandt in 1994, have anterior chamber deepening, pupil dilation, posterior iris bowing, and discomfort during phacoemulsification [15]. This syndrome is caused by a reverse pupillary block, possibly as a result of weak ciliary muscle and elongated zonular fibers, and it occurs more often in myopic eyes and in previously vitrectomized eyes that are more susceptible to excessive deepening of the anterior chamber [16, 17]. In our study, patients with greater AL and larger ACD were more susceptible to pain, supporting the idea that eyes suffering from lens-iris diaphragm retropulsion syndrome tend to have significant discomfort during phacoemulsification under topical anesthesia.

Patients who had higher preoperative IOP, larger ACD, and/or greater AL experiencing more surgical pain and additional strategies for reducing pain should be considered in these cases. There are two strategic approaches for relieving surgical pain. One is adding another anesthetic or analgesic treatment, and the other strategy is reducing lens-iris diaphragm fluctuation.

The use of intracameral lidocaine can be effective to relieve discomfort, especially during iris manipulation, although its use is controversial. Studies showed that the use of supplementary intracameral lidocaine injection with topical anesthesia is an effective and safe adjunct for decreasing intraoperative pain [18, 19], whereas another study reported there was no significant difference when using additional intracameral anesthesia [20]. This procedure might be necessary according to the needs of the surgeon or patient.

Topical nonsteroidal anti-inflammatory drugs (NSAIDs) also can be an alternative option in these patients. According to Price and Price, applying ophthalmic ketorolac 0.4% solution for 3 days prior to and 1 day following surgery was an effective treatment to control discomfort associated with cataract surgery [21]. In addition to providing pain control, topical NSAIDs are effective for postoperative inflammation control and preventing intraoperative miosis and postoperative cystoid macular edema in cataract surgery patients despite corneal complications. Thus, proper use of topical NSAIDs also could be an alternative to relieve patient discomfort.

Surgical techniques or modification of surgical parameters to avoid overextension of the anterior chamber were necessary in patients with larger ACD or greater AL. Management options include reducing the height of the infusion bottle and adding a second infusion line to relieve overextension of the anterior chamber by decreasing the infusion pressure [15]. Intraoperative use of a flexible iris retractor could be an option to relieve the reverse pupillary block. By doing this, the iris can be lifted continuously from the anterior capsule rim and held securely from the wound and phacoemulsification probe to minimize the chance of iris prolapsed and damage [22]. The technique enlarging the existing continuous curvilinear capsulorhexis also could be considered to avoid iris-capsule contact and to relieve the reverse pupillary block [23].

We designed this study to use topical anesthesia alone and did not use additional anesthesia, as we thought it unnecessary. However, the mean pain scores were higher than those reported in other researches. However, all patients in those studies also received additional preoperative intravenous or oral sedation, which likely explains the discrepancy.

In summary, we found that patients with higher preoperative IOP, larger ACD, and/or greater AL experienced more pain during and after phacoemulsification under topical anesthesia alone. Although the use of topical anesthesia is safe in cataract surgery, patients should be informed about the perceived pain preoperatively, and additional procedures can be used as needed to relieve discomfort and to improve cooperation during surgery and postoperatively.

## Figures and Tables

**Table 1:** Standard surgical parameters using the Infiniti Vision System.

Group	Mode	Fluid height (cm)	Aspiration flow (mL/min, mode)	Vacuum (mmHg, mode)
Low parameter	Sculpting	70	22, linear	90, fixed
	Chopping & quadrant	70	27, linear	240, linear
High parameter	Sculpting	90	22, linear	90, fixed
	Chopping & quadrant	110	38, linear	380, linear

**Table 2:** Baseline characteristics of patients undergoing phacoemulsification.

Characteristics	Value
Number of eyes, *n* (%)	
OD	68 (47.9%)
OS	74 (52.1%)
Sex, *n* (%)	
Male	70 (49.3%)
Female	72 (50.7%)
Age, years	67.49 ± 12.50
Underlying disease, *n* (%)	
Diabetes mellitus	40 (28.2%)
Hypertension	45 (31.7%)
Benign prostatic hyperplasia	6 (4.2%)
Retinal disease	28 (19.7%)
Glaucoma	32 (22.5%)
Anterior chamber depth, mm	3.09 ± 0.39
Axial length, mm	23.77 ± 1.76
Intraocular pressure, mmHg	14.46 ± 3.10
LogMAR BCVA	0.70 ± 0.63
Central corneal thickness, *μ*m	432 ± 228
Endothelial cell density, cells/mm^2^	2631 ± 483
Nuclear cataract grading	3.69 ± 1.25

Values are presented as the mean ± SD. BCVA: best-corrected visual acuity.

**Table 3:** Intraopeartive parameters.

Characteristics	Value
Operation time, min	21.44 ± 3.18
Ultrasound time, sec	16.53 ± 30.84
Cumulative dissipated energy	17.79 ± 1.22
Mean volume of BSS, mL	91.88 ± 50.86
Parameter of phacoemulsification, *n* (%)	
Low parameter	134 (94.4%)
High parameter	8 (5.6%)

Values are presented as the mean ± SD. BSS: balanced salt solution.

**Table 4:** Univariate and multivariate analyses of predictors affecting intraoperative pain during phacoemulsification.

Parameters	Univariate			Multivariate		
	*β* coefficient	CI (95%)	*P* value	*β* coefficient	CI (95%)	*P* value
Mean age	−0.130	−0.020 to 0.002	0.123	—	—	—
Male sex	0.037	−0.219 to 0.345	0.659	—	—	—
Site of operation	0.029	−0.234 to 0.331	0.735	—	—	—
Log BCVA	0.100	−0.090 to 0.359	0.238	—	—	—
Mean preoperative IOP	0.225	0.017 to 0.107	0.007	0.220	0.012 to 0.118	0.016
Mean AL	0.255	0.045 to 0.201	0.002	0.181	−0.005 to 0.179	0.043
Mean ACD	0.202	0.083 to 0.790	0.016	0.210	0.052 to 0.883	0.028
Mean CCT	0.035	−0.005 to 0.004	0.829	—	—	—
Mean nuclear cataract grading	−0.185	−0.238 to −0.014	0.028	−0.123	−0.242 to 0.075	0.298
Mean endothelial cell count	−0.098	−0.001 to 0.000	0.245	—	—	—
Mean operation time	0.086	−0.021 to 0.068	0.306	—	—	—
Mean ultrasound time	−0.133	−0.008 to 0.001	0.115	—	—	—
Mean CDE	−0.083	−0.015 to 0.005	0.328	—	—	—
Mean volume of BSS	−0.132	−0.005 to 0.001	0.117	—	—	—
Standard surgical parameter (low versus high surgical parameters)	−0.130	−0.838 to 0.101	0.123	—	—	—
Use of intracameral epinephrine	0.058	−0.196 to 0.403	0.497	—	—	—
Use of intracameral carbachol	−0.519	−1.125 to 0.087	0.093	—	—	—
Use of capsular tension ring	0.103	−0.279 to 0.486	0.594	—	—	—

ACD: anterior chamber depth; AL: axial length; BCVA: best-corrected visual acuity; BSS: balanced salt solution; CCT: central corneal thickness; CDE: cumulative dissipated energy; CI: confidence interval; IOP: intraocular pressure.
